# Glutathione and Ascorbic Acid Accumulation in Mango Pulp Under Enhanced UV-B Based on Transcriptome

**DOI:** 10.3390/antiox13111429

**Published:** 2024-11-20

**Authors:** Hassam Tahir, Muhammad Sajjad, Minjie Qian, Muhammad Zeeshan Ul Haq, Ashar Tahir, Muhammad Aamir Farooq, Ling Wei, Shaopu Shi, Kaibing Zhou, Quansheng Yao

**Affiliations:** 1 Sanya Institute of Breeding and Multiplication, Hainan University, Sanya 572025, China; 2 School of Tropical Agriculture and Forestry, Hainan University, Haikou 570228, China; 3 College of Ecology and Environment, Hainan University, Haikou 570228, China; 4 Key Laboratory for Postharvest Physiology and Technology of Tropical Horticultural Products of Hainan Province, Zhanjiang 524091, China

**Keywords:** *Mangifera indica* (Tainong 1), enhanced UV-B radiation, glutathione, ascorbic acid, transcriptome

## Abstract

Mango (*Mangifera indica*), a nutritionally rich tropical fruit, is significantly impacted by UV-B radiation, which induces oxidative stress and disrupts physiological processes. This study aimed to investigate mango pulp’s molecular and biochemical responses to UV-B stress (96 kJ/mol) from the unripe to mature stages over three consecutive years, with samples collected at 10-day intervals. UV-B stress affected both non-enzymatic parameters, such as maturity index, reactive oxygen species (ROS) levels, membrane permeability, and key enzymatic components of the ascorbate-glutathione (AsA-GSH) cycle. These enzymes included glutathione reductase (GR), gamma-glutamyl transferase (GGT), glutathione S-transferases (GST), glutathione peroxidase (GPX), glucose-6-phosphate dehydrogenase (G6PDH), galactono-1,4-lactone dehydrogenase (GalLDH), ascorbate peroxidase (APX), ascorbate oxidase (AAO), and monodehydroascorbate reductase (MDHAR). Transcriptomic analysis revealed 18 differentially expressed genes (DEGs) related to the AsA-GSH cycle, including *MiGR*, *MiGGT1*, *MiGGT2*, *MiGPX1*, *MiGPX2*, *MiGST1*, *MiGST2*, *MiGST3*, *MiG6PDH1*, *MiG6PDH2*, *MiGalLDH*, *MiAPX1*, *MiAPX2*, *MiAAO1*, *MiAAO2*, *MiAAO3*, *MiAAO4*, and *MiMDHAR*, validated through qRT-PCR. The findings suggest that UV-B stress activates a complex regulatory network in mango pulp to optimize ROS detoxification and conserve antioxidants, offering insights for enhancing the resilience of tropical fruit trees to environmental stressors.

## 1. Introduction

UV-B is one of the abiotic stressors that directly impacts the growth and development of plants [1]. UV-B triggers a range of physiological, biochemical, metabolic, and molecular changes in plants, leading to heightened oxidative stress, which in turn hampers plant growth and metabolism [2]. Enhanced UV-B radiation (280–315 nm) from ozone depletion adversely impacted the environment, affecting plant morphology, physiology, and processes [3]. It inhibits plant growth, reduces leaf size, height, and biomass, and alters flowering patterns [4]. The annual rise rate of enhanced UV-B radiation in the northern and southern hemispheres is expected to reach approximately 14% and 40%, respectively, raising concerns about the risks associated with enhanced UV-B radiation [5].

Enhanced UV-B radiation stress induces the production of reactive oxygen species (ROS), causing oxidative damage to cells and disrupting biochemical and physiological functions, thereby affecting plant growth and metabolism [6,7]. UV-B radiation increases ROS production, including hydrogen peroxide (H_2_O_2_), singlet oxygen (1O_2_•), superoxide anion (O_2_•^−^), and hydroxyl radicals (•OH) [8]. To combat the detrimental effects of ROS, plants have developed a robust intracellular antioxidant defense system comprising both enzymatic and non-enzymatic antioxidant mechanisms [9,10]. Enzymatic antioxidants include glutathione peroxidase (GPX; EC:1.11.1.9), ascorbate peroxidase (APX; EC:1.11.1.11), monodehydroascorbate reductase (MDHAR; EC:1.6.5.4), glutathione reductase (GR; EC:1.8.1.7), and glutathione S-transferases (GST; EC:2.5.1.18) [11]. Non-enzymatic antioxidants include ascorbic acid and glutathione [12]. The AsA-GSH cycle involving APX, MDHAR, and GR, is a highly effective pathway for H_2_O_2_ detoxification [13]. Maintaining this balance is critical for plant health, as imbalances can lead to oxidative damage to lipids, proteins, and nucleic acids [14]. Ascorbic acid (AsA) is crucial for UV-B tolerance; its deficiency leads to hypersensitivity [15]. AsA and GSH help maintain homeostasis and protect membranes under high UV-B exposure, influencing protective pathways [16,17]. GSH boosts antioxidant capacity in strawberries and protects them from abiotic stress by scavenging H_2_O_2_ through the AsA-GSH cycle [18]. Recent advances have identified key enzymes and genes involved in AsA metabolism in fruit, such as *PbrAPX8/10* and *PbrAO3* in ‘Yali’ pears [19]. Enzymes like glucose-6-phosphate 1-dehydrogenase (G6PDH; EC:1.1.1.49) generate NADPH, which is essential for the AsA-GSH cycle and plays a crucial role in ROS scavenging [20]. Additionally, GPX and GST are important for reducing H_2_O_2_ in plant tissues by converting GSH to its oxidized form (GSSG), protecting membranes from oxidative damage [21]. In Arabidopsis, the *AtGPX8* gene is known for safeguarding cells against oxidative stress [22]. Kato and Esaka also suggest that ascorbate oxidase (AAO; EC:1.10.3.3) regulates cell expansion by influencing plasma membrane transport and participates in stress responses by modulating ascorbic acid levels and redox balance, enabling plants to respond to environmental cues effectively [23,24].

Mango (*Mangifera indica*), commonly referred to as the “king of tropical fruits”, is an important cash crop belonging to the Anacardiaceae family. It holds the position of the fifth most widely produced fruit globally. Mangoes are rich in nutrients such as bioactive compounds, proteins, vitamins, carbohydrates, lipids, and organic acids [25,26]. In addition to being consumed raw, they can be processed into a wide range of goods, including powder, nectar, jam, juice, and jellies [27]. China is the second-largest mango producer globally, with key cultivation areas in Sichuan, Hainan, Yunnan, and Guangxi [28]. Mangoes have a domestication history of over 4000 years in the Indo-Burmese and Southeast Asian regions and have spread worldwide since the 14th century [29]. Despite abiotic and biotic challenges, mangoes remain an important source of income [30,31].

Enhanced UV-B radiation, particularly in tropical regions, significantly affects mango trees, harming leaves, chloroplasts, and photosynthetic tissue, thus reducing yield and fruit quality. Although mango naturally tolerates UV-B radiation well, prolonged exposure worsens ROS damage due to decreased enzyme activity and pigment levels [32,33]. Limited research has been conducted on the impact of heightened UV-B radiation on mango pulp, particularly concerning pathways involving GSH and AsA. This study aims to investigate the changes in enzymatic and non-enzymatic antioxidants, GSH and AsA levels, and the regulatory mechanisms by which UV-B influences the biosynthesis of GSH and AsA through transcriptome analysis. These findings are anticipated to establish foundational insights into the effects of UV-B application, offering essential data for understanding the mechanisms underlying mango adaptation to increased UV-B exposure. Furthermore, this research may provide practical guidance for developing sustainable cultivation practices for mangoes under enhanced UV-B conditions while also serving as a reference for studying UV-B stress resistance mechanisms in other crops.

## 2. Materials and Methods

### 2.1. Experimental Site and Mango Phenotypic Observation

The research took place in Hainan Province, Sanya City, Haitong District, and Shengchang Village (18°25′ N, 109°46′ E), within a 15-year-old mango orchard of the “Tainong 1” variety, which had been grafted onto “Changjiang Tumang” rootstock. The usual yearly rainfall in the region is approximately 1700 mm, and the standard temperature is approximately 25 °C. The soil of the plot of land is categorized as brick-red sandy. Growth time amendment technology was employed in the later month of July to guarantee a mango supply for the Chinese Spring Festival. The major growth phases were observed during the 2021–2022 cycle: flower bud development took place from August to September, with bud emergence occurring in early October. Blooming occurred between mid and late October, with physiological flower and fruit dropping in early to mid-November. The fruit expansion phase spanned from December to January, and the harvest occurred in early to mid-February. In the 2022–2023 cycle, each stage occurred about a month earlier, and by the 2023–2024 cycle, these stages advanced by approximately half a month, reflecting the orchard’s ongoing adaptation in its production cycle.

### 2.2. Soil Physiochemical Properties and Weather Patterns Including Natural UV Levels

The experimental garden soil was fertile latosol, with the following physicochemical properties measured at the beginning of the study in 2021: soil organic matter (SOM) was 23.52 ± 0.74 g kg^−1^, available nitrogen (AN) was 97.50 ± 6.23 mg kg^−1^, available phosphorus (AP) was 43.64 ± 6.15 mg kg^−1^, and available potassium (AK) was 125.63 ± 7.46 mg kg^−1^. The area receives an average annual precipitation of approximately 1700 mm, with an average yearly temperature of 23.8 °C. The monthly maximum temperature can reach around 35 °C, while the minimum is typically above 10 °C. The average annual sunshine duration is approximately 2100 h. The UV-B radiation intensities during the sampling periods for each production season are provided in Table S1.

### 2.3. Treatment of UV-B Radiation

An aluminum alloy UV lamp stand was installed for the field experiment, with natural light averaging 600 kJ·m^−2^·d^−1^. Four 40 W UV lamps, sourced from the Beijing Electric Light Source Research Institute, were vertically suspended 30 cm above the tree apex. These lamps emitted light at 313 nm with an intensity of 96 kJ·m^−2^·d^−1^, simulating high-dose UV-B radiation equivalent to a 15% increase in UV-B exposure. The natural sunrise-to-sunset cycle operates the lamps, with intervals during rain to replicate natural solar radiation. UV-B intensity remained stable at approximately 24 kJ·m^−2^·d^−1^ at 30 cm below the lamps. Ten trees were selected for this study, and sampling was conducted every ten days, including control, using five vigorous, homogeneously shaped fruits from the outer intermediate canopy of a singlet tree plot, which was used for biological replicates. Each sampling interval included three replicates, and each replicate had five fruits. The sampling periods were as follows: 30 to 90 d after flowering during the first season (18 December to 17 February 2022); 40 to 90 d after flowering in the second season (1 December 2022, to 21 January 2023); and 30 to 90 d after flowering in the third season (6 November 2023, to 6 January 2024), adjusted by 10 d to align with Spring Festival demand. Samples were processed in the orchard and stored at −80 °C for future analysis. Physiological and biochemical indicators were assessed using samples from the first two seasons, including fruit ripening index, ROS levels, lipid peroxidation, ion leakage, glutathione content, ascorbic acid levels, and enzymatic activity of key antioxidant pathways. RNA-seq analysis was conducted on samples from the third season.

### 2.4. Fruit Ripening Index

The fruit ripening index, which included measurements of total soluble solids (TSS) and titratable acidity (TA), was determined by a digital refractometer (RX 5000, ATAGO, Tokyo, Japan). To ensure accuracy, the prism of the refractometer was carefully cleaned with ethanol before placing the juice sample. After placing the sample, the lid was gently closed, and the measurements were recorded in degrees Brix (°Brix) at room temperature [34]. The TSS-to-TA ratio was subsequently calculated by dividing the total soluble solids (°Brix) by the percentage of titratable acidity (% Acid).

### 2.5. Lipid Peroxidation

Lipid peroxidation levels were evaluated following the method outlined by Ding et al. [35]. At first, 1.00 g of the sample was carefully prepared in 5% trichloroacetic acid (TCA), ensuring a consistent homogenate. This mixture was then centrifugated to separate the solid components, leaving a clear supernatant for further analysis. Next, the supernatant was combined with an equal volume of 5% TCA, which contained 0.67% thiobarbituric acid. The mixture was heated to promote the reaction and cooled to stabilize the complex. The absorbance of the final solution was measured at multiple wavelengths, including 532 nm, 600 nm, and 450 nm, providing the necessary data to calculate malondialdehyde (MDA). The MDA concentration was derived using the following equation: MDA = [6.45 (OD532 − OD600) − 0.560 × OD450].

### 2.6. Ion Leakage

Ion leakage was evaluated following the protocol outlined by Khaliq et al. [36], with slight modifications to adapt to the specific conditions of this experiment. A 5.00 g sample was gently placed into a glass tube, then 25 mL of deionized water was added. The sample was subjected to gentle agitation at a rate of 1.7 s^−1^ for 30 min, maintained at a consistent temperature of 24 °C. A DDS-11A conductivity meter (Shanghai, China) was used to measure the initial conductivity. The glass tube containing the sample was heated in a water bath set at 98 °C for 15 min. After heating, the sample was cooled to normal temperature, and a second conductivity reading was taken using the same meter. The extent of ion leakage from the sample was then calculated by applying the following formula: relative conductivity (RC) was determined as the ratio of the initial conductivity (S1) to the final conductivity (S2), expressed as RC = (S1/S2) × 100%.

### 2.7. Detection of ROS

To measure the contents of H_2_O_2_, •OH, and O_2_^−^ in the samples, specialized assay kits were employed. These kits, sourced from Jiangsu Kete Biotechnology Co., Ltd. (Yancheng City, Jiangsu province, China), were selected to ensure precision and reliability in the detection process. The kits used included Catalog No. ADS-W-YH001 for hydrogen peroxide, ADS-W-KY006 for hydroxyl ion, and ADS-W-YH008 for superoxide anion.

### 2.8. Enzymatic Activity Assessment

The extraction and activity assays of enzymes AAO, G6PDH, GST, GGT, APX, GPX, GR, GalLDH, and MDHAR from fresh mango pulp samples were conducted using assay kits from Comin Suzhou Keming Biotechnology Co., Ltd. (Suzhou, China). For each enzyme, 0.10 g of sample was ground with 1 mL of the respective extraction reagent, followed by centrifugation under specific conditions: GR and MDHAR at 10,000 rpm for 10 min, GGT at 8000× *g* for 15 min, GST and G6PDH at 8000× *g* for 10 min, GPX and APX at 12,000× *g* for 10 min and 20 min, GalLDH at 13,000× *g* for 10 min, and AAO at 16,000× *g* for 10 min. The supernatants obtained were then used to assess enzyme activities. The enzyme activities were measured based on specific reactions: AAO activity was determined by measuring the oxidation of ascorbate at 265 nm, where one enzyme activity unit was defined as 1 nmol of protein, representing the AAO enzyme activity that oxidizes ascorbate per minute of protein (Catalogue No. AAO-2-W). G6PDH activity was measured by observing the reduction of NADP+ to NADPH at 340 nm; one enzyme activity unit was defined as the production of 1 nmol NADPH of enzyme activity per minute (Catalog No. G6PDH-2-Y). GST activity was assessed by catalyzing GSH and CDNB at 340 nm, where one enzyme activity unit was defined as 1 nmol of the enzyme activity catalyzing this reaction per minute (Catalog No. GST-2-W). GGT activity was determined by measuring the catalyzed gamma-glutamyl group in glutamyl-p-nitroaniline to N-glycylglycine at 405 nm; one enzyme activity unit was defined as the production of 1 nmol p-nitroaniline of enzyme activity per minute (Catalog No. GGT-2-W). APX activity was determined by the analysis of the AsA oxidation rate at 290nm; one enzyme activity unit was defined as the oxidation of 1 nmol of ascorbate per minute (Catalog No. APX-2-W). GPX activity was evaluated by tracking the oxidation of NADPH at 340 nm, with one enzyme activity unit defined as 1 nmol of protein representing enzyme activity per minute (Catalog No. GPX-2-W). GR activity was determined by the measurement of the dehydrogenation rate of NADPH at 340 nm; one enzyme activity unit was defined as the oxidation of 1 nmol representing enzyme activity per minute (Catalog No. GR-2-W). GalLDH activity was determined through the measurement of the increment rate of reduced Cyt c at 550 nm; one enzyme activity unit was defined as the production of 1 μmol reduced Cyt c enzyme activity per minute (Catalog No. GLDH-2-W). Lastly, MDHAR activity was determined by the measurement of the reduction rate of NADH at 340 nm; one enzyme activity unit was defined as the oxidation of 1 nmol of enzyme activity per minute (Catalog No. MDHAR-2-W). The BCA protein concentration assay kit (Catalogue No. BL521A available from Biosharp; http://www.biosharp.cn/) evaluated soluble proteins in mango pulp samples. The samples (0.10 g) were first homogenized in 2 mL of cold phosphate-buffered saline and centrifuged at 12,000× *g* for 10 min. After centrifugation, we took 20 μL of supernatant and then added 200 μL BCA working liquid (2.0 mg. mL^−1^ in 75 mM phosphate buffer, pH 7.4) to the microplate, mixed them well, and incubated them at 37 °C for 30 min. The absorbance was then measured at 562 nm.

### 2.9. Glutathione Content

GSH content was measured using the GSH Measurement Kit (GSH-1-W, Suzhou Keming Biotechnology Co., Ltd., Suzhou, China) according to the manufacturer’s instructions. As outlined in the kit protocol, we homogenized 0.2 g of frozen plant material for each sample. After preparation, UV transmission density was measured at 412 nm for both the test samples and a blank control. The absorbance of the blank solution (A2) and each sample (A1) was recorded. The difference between these values (ΔA = A2 − A1) was calculated to account for background interference, and this value was used to determine the GSH content. The glutathione concentration was then calculated using the formula GSH content (µmol g^−1^) = 0.667 × (A2 − A1)/W, where W represents the sample weight in grams.

### 2.10. AsA Content

Ascorbic acid content was determined using the 2,6-dichlorophenol indophenol (DCPIP) titration method, following the procedure described by Rao and Deshpande [37]. The procedure involved preparing a working standard of ascorbic acid at a concentration of 500 µg in 5 mL. In a 100 mL conical flask, 5 mL of this standard solution was combined with 10 mL of 4% oxalic acid to create the standard titration mixture. Titration was performed by gradually adding the DCPIP dye solution until a persistent faint pink color was observed, indicating the end point. The volume of dye needed to reach this end point was recorded as V1. The same procedure was applied to the test sample, with 5 mL of the sample solution being titrated with the DCPIP dye, and the volume of dye used for this titration was recorded as V2.

### 2.11. RNA Extraction and Detection

Frozen pulp samples were collected at 30, 40, and 90 days from the third season, with three biological replicates for each time point. Total RNA was extracted from these samples using the CTAB-PBIOZOL method. After extraction, RNA quantity and quality were evaluated using a Qubit fluorescence quantifier and a Qsep400 high-throughput fragment analyzer to ensure sample integrity and purity. Transcriptome sequencing was performed on the Illumina HiSeq platform. Raw sequencing reads were processed with Fastq software (v0.18.0) [38] to remove low-quality sequences and adapters, retaining only high-quality clean reads for further analysis. The quality-controlled reads were aligned to the mango reference genome (available at https://www.ncbi.nlm.nih.gov/genome/?term=mango; accessed on 2 September 2024) using HISAT2 (v2.2.0) [39], an efficient RNA-seq alignment tool. Mapped reads were assembled using StringTie (v1.3.1) [40], which uses a reference-based approach to reconstruct transcripts. Gene expression levels were quantified by calculating alignment statistics with feature counts, and expression variation and abundance for each gene were analyzed. The Fragments Per Kilobase Million (FPKM) values for each gene were calculated using StringTie, providing detailed gene expression measurements across the different time points.

### 2.12. Differential and Enrichment Analysis

Gene differential expression analysis between two groups was carried out using DESeq2 software (v3.20) [41], applying a threshold of absolute fold change >1 and a false discovery rate (FDR) < 0.05. To gain insights into the biological functions of the differentially expressed genes (DEGs), Gene Ontology (GO) enrichment analysis was performed, categorizing the DEGs into functional groups [42]. Each DEG was mapped to the GO database (http://www.geneontology.org) to identify relevant functional terms. A hypergeometric test was used to detect GO terms significantly enriched in the DEGs by comparing the distribution of GO terms in the DEGs to those in the reference genome. Pathway enrichment analysis was conducted using the Kyoto Encyclopedia of Genes and Genomes (KEGG) database (https://www.genome.jp/kegg/) [43], which helped identify key metabolic and signaling pathways. *p*-values from the enrichment analyses were adjusted using the false discovery rate (FDR) correction method to address multiple testing issues. Pathways with an FDR ≤ 0.05 were considered significantly enriched.

### 2.13. qRT-PCR Analysis

To validate the RNA-seq results, eighteen differentially expressed genes (DEGs) from the AsA-GSH pathways were selected for quantitative real-time PCR (qRT-PCR) analysis. Primers for the qRT-PCR were designed using PrimerPremier 6 software and synthesized by Shanghai Bioengineering Co., Ltd. (Shanghai, China) (Table S2). Total RNA from each sample was converted into cDNA using the HiScript II First-Strand cDNA Synthesis Kit (Novizan Biotechnology Co., Ltd., Nanjing, China) in a T100™ Thermal Cycler (Bio-Rad, Hercules, CA, USA). qRT-PCR was conducted with the qTOWER^3^ QPCR system (Analytik Jena AG, Jena, Germany) using Tolo Biotech 2× Q3 SYBR qPCR Master Mix, according to the manufacturer’s instructions. Reactions were performed in 96-well plates, with Actin used as an internal reference gene for normalization. Gene expression levels were determined using the 2^−ΔΔCt^ method [44], facilitating a reliable comparison between the selected DEGs and the RNA-seq data.

### 2.14. Statistical Analysis

Data analysis was conducted using SAS 9.4 statistical software (SAS Institute Inc., Cary, NC, USA). The study employed a randomized complete block design (RCBD) and used two-way analysis of variance (ANOVA) to evaluate variance in dynamic changes across experimental groups. The Tukey HSD test was applied to determine statistically significant differences for multiple comparisons at various time points. Graphical data representations were created using GraphPad Prism 8.0.1 to provide clear visualizations. Additionally, a heatmap of the expression patterns of differentially expressed genes (DEGs) was generated with TBtools-II software (v2.096) [45].

## 3. Results

### 3.1. Maturity Index in Mango Pulp Under UV-B Stress

**Figure 1:**
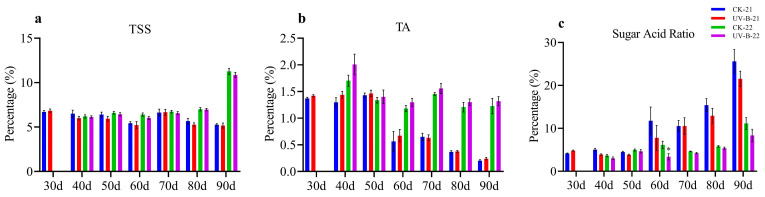
The graphs depict the changes in mango fruit pulp quality over the 2021–2022 and 2022–2023 growing seasons, focusing on key parameters: (**a**) TSS, (**b**) TA, and (**c**) TSS/TA. Significant differences between the control (CK) and UV-B treatment groups are marked with * for *p* < 0.05.

The influence of UV-B treatment on fruit quality, specifically total soluble solids (TSS), titratable acidity (TA), and the sugar-to-acid ratio, was assessed over two years (2021–2022 and 2022–2023). As shown in Figure 1a, TSS levels in both treated and control groups decreased over time in the first year, while the TSS remained constant except for 90 d (10.86) in the second year, but there is no significant difference between treatment and control. Similarly, Figure 1b indicated that the TA level showed a decreasing trend, where it decreased after 60 d (0.67) in the first year and gradually decreased after 40 d (2.0) in the second year. However, there were no significant differences between the treatment and control. Figure 1c shows that the sugar-to-acid ratio reduced at 80 d and 90 d (12.92 and 21.52) in the first year and showed a significant decrease at 60 d (3.37) in the second year in UV-B-treated samples compared to the control. These results highlight the effects of UV-B treatment on fruit quality, particularly in regulating sugar and acidity during ripening.

### 3.2. Membrane Permeability and ROS in Mango Pulp Under UV-B Stress

**Figure 2:**
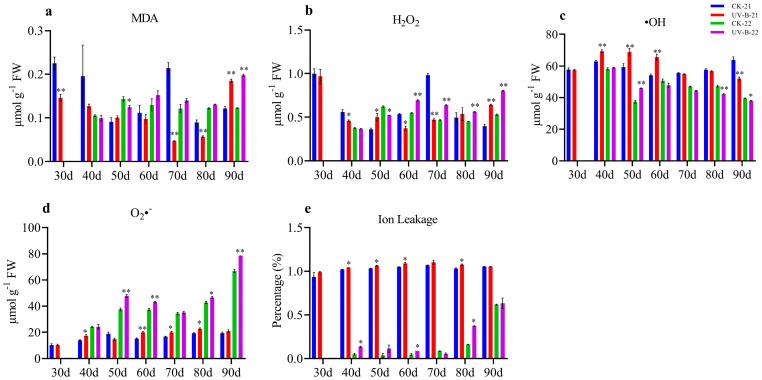
The dynamic changes in mango fruit pulp during the 2021–2022 and 2022–2023 growing seasons are depicted in the graphs, showcasing key oxidative stress markers and physiological parameters: (**a**) MDA, (**b**) H_2_O_2_, (**c**) O_2_•^−^, (**d**) •OH, and (**e**) ion leakage. At each time point, significant differences between the control (CK) and UV-B treatment groups are indicated by * for *p* < 0.05 and ** for *p* < 0.01, respectively.

The analysis of membrane stability, relative conductivity, and oxidative stress markers under UV-B treatment during the 2021–2022 and 2022–2023 growing seasons revealed significant physiological changes in mango fruit pulp. Figure 2a shows a notable increase in MDA levels in UV-B-treated samples compared to the control, especially at later growth stages, with a significant rise observed at 90 d in both years (0.18 and 0.19). However, MDA levels were lower at 30, 70, and 80 d (0.14, 0.04, and 0.05) in the first year and at 50 d (0.12) in the second year, with no significant differences at other time points. Figure 2b shows that H_2_O_2_ levels also increased significantly under UV-B treatment as compared to the control at 50 and 90 d (0.50 and 0.64) in the first year and at 60 and 90 d (0.69 and 0.80) in the second year, However, H_2_O_2_ levels decreased at 40, 60, and 70 days (0.45, 0.37, and 0.46) in the first year and at 50 d (0.52) in the second year. Figure 2c depicts fluctuating •OH levels, with significant increases at 40, 50, and 60 d (69.32, 68.85, and 65.65) in the first year and at 50 d (45.97) in the second year, but showed a decreased trend at 90 d (51.99) in the first year and at 80 and 90 d (42.26 and 38.05) in the second year. Figure 2d reveals a significant increase in O_2_•^−^ levels under UV-B treatment, with substantial rises at 40, 60, 70, and 80 d (17.24, 19.83, 19.88, and 22.79) in the first year and at 50, 60, 80, and 90 days (47.71, 43.05,46.54, and 78.44) in the second year. The O_2_•^−^ content in UV-B-treated samples remained significantly higher than in the control. Figure 2e illustrates a considerable increase in ion leakage under UV-B treatment, with notable rises at 40, 50, 60, and 80 days (1.04, 1.06, 1.08, and 1.07) in the first year and at 40, 60, and 80 d (0.13, 0.08 and 0.37) in the second year, where it remained higher than the control. These findings indicate that UV-B treatment induces oxidative stress and affects membrane stability, leading to significant physiological changes throughout fruit development.

### 3.3. Non-Enzymatic Antioxidants in Mango Pulp Under UV-B Stress

**Figure 3:**
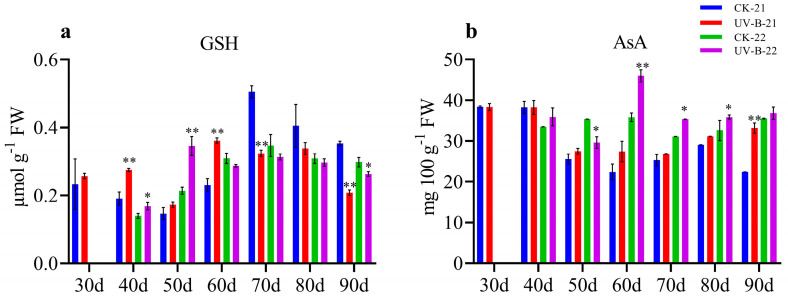
The graphs illustrate the fluctuations in antioxidative parameters in mango fruit pulp during the 2021–2022 and 2022–2023 growing seasons: (**a**) GSH levels and (**b**) AsA levels. At each time point, significant differences between the control (CK) and UV-B treatment groups are indicated by * for *p* < 0.05 and ** for *p* < 0.01.

The analysis of GSH and AsA levels in mango pulp exposed to UV-B stress during the 2021–2022 and 2022–2023 growing seasons revealed important insights into the fruit’s antioxidative responses. Figure 3a shows that GSH levels in the UV-B-treated mango pulp exhibited a fluctuating pattern compared to the control. Specifically, GSH content increased significantly at 40 and 60 d (0.27 and 0.36) in the first year and at 50 d (0.34) in the second year. However, GSH levels decreased at 70 and 90 d (0.32 and 0.20) in the first year and at 90 d (0.26) in the second year. Overall, the GSH content in the UV-B treatment group was notably higher than in the control group before 70 d but declined afterward. Similarly, Figure 3b demonstrates that AsA content in UV-B-treated samples was significantly higher at 80 and 90 d (31.09 and 33.16) in the first year and at 60 to 80 d (46, 35.34, and 35.85) in the second year compared to the control. Nonetheless, a significant drop in AsA content was observed at 50 d (29.59) in the second year. The AsA levels in the treatment group were substantially higher than those in the control group across both years from 60 to 90 d, with no significant differences noted at other time points. These findings underscore the dynamic antioxidative response of mango fruit to UV-B stress, with varying levels of GSH and AsA reflecting the plant’s adaptive strategies to manage oxidative damage.

### 3.4. Enzymatic Activity of GSH in Mango Pulp Under UV-B Stress

**Figure 4:**
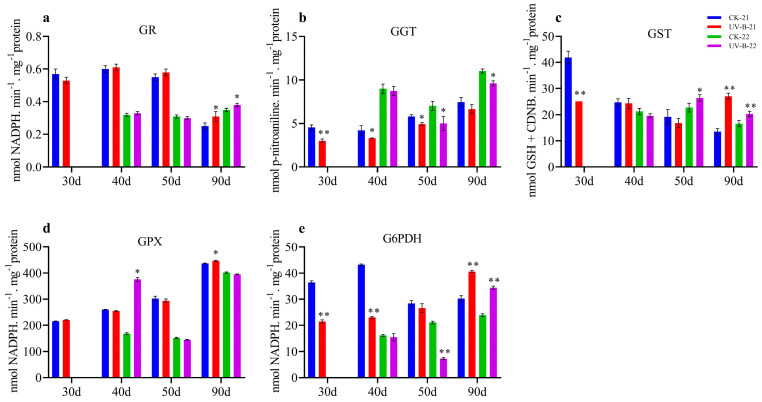
The dynamic changes in GSH enzymatic activity in mango fruit pulp during the 2021–2022 and 2022–2023 growing seasons are illustrated in the following graphs: (**a**) GR, (**b**) GGT, (**c**) GST, (**d**) GPX, and (**e**) G6PDH. Significant differences between the control (CK) and UV-B treatment groups at each time point are denoted by * for *p* < 0.05 and ** for *p* < 0.01, respectively.

This study examined the impact of UV-B treatment on various enzymatic activities related to oxidative stress and antioxidant defenses in mango fruit over two years (2021–2022 and 2022–2023). The results revealed significant differences between the UV-B-treated and control groups, highlighting UV-B’s pronounced effect on enzymatic activities. Specifically, UV-B treatment led to a notable increase in GR activity at 90 d in both years (0.31 and 0.38), as depicted in Figure 4a. Conversely, GGT activity decreased significantly at 30, 40, and 50 d (3.0, 3.31, 4.94) in the first year and at 50 and 90 d (5.0 and 9.62) in the second year for UV-B-treated samples, as shown in Figure 4b. Additionally, GST activity increased at 90 d (27.07) in the first year and 50 and 90 d (26.38 and 20.35) in the second year, although it was lower at 30 d in the first year (Figure 4c), while GPX activity rose at 90 d (446.5) in the first year and at 40 d (375.91) in the second year (Figure 4d). Glucose-6-phosphate dehydrogenase (G6PDH) activity was significantly elevated at 90 d (40.61 and 34.35) in both years, although it was lower at 30 and 40 d (21.53 and 23.05) in the first year and at 50 d (7.35) in the second year (Figure 4e). Overall, the UV-B treatment markedly affected enzymatic activities linked to oxidative stress responses and antioxidant defenses in mango fruit.

### 3.5. Enzymatic Activity of AsA in Mango Pulp Under UV-B Stress

**Figure 5:**
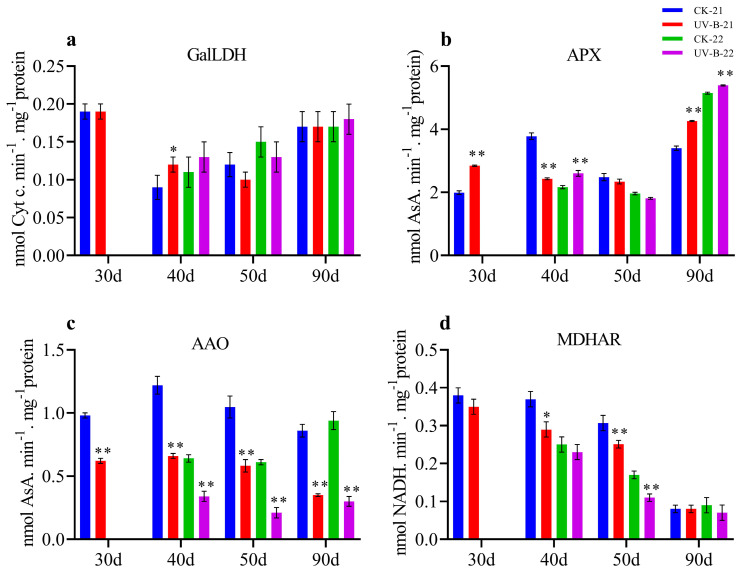
The graphs below depict the variations in AsA-related enzymatic activity in mango fruit pulp over the 2021–2022 and 2022–2023 growing seasons: (**a**) GalLDH, (**b**) APX, (**c**) AAO, and (**d**) MDHAR. Significant differences between the control (CK) and UV-B treatment groups at each time point are marked with * for *p* < 0.05 and ** for *p* < 0.01.

This study investigated the impact of UV-B treatment on enzymatic activities related to AsA, including GalLDH, APX, AAO, and MDHAR over two consecutive years (2021–2022 and 2022–2023). The results revealed notable differences between the UV-B-treated and control groups, emphasizing the significant effect of UV-B radiation on these enzymes. Figure 5a shows a marked increase in GalLDH activity in UV-B-treated pulp, with substantial peaks at 40 d (0.12) in the first year. Similarly, Figure 5b demonstrates an increase in APX activity in UV-B-treated samples at 30 and 90 d (2.84 and 4.26) in the first year and at 40 and 90 d (2.6 and 5.39) in the second year. However, APX activity was lower at 40 d (2.43) in the first year. In contrast, Figure 5c shows a significant reduction in AAO activity in UV-B-treated samples at 30, 40, 50, and 90 d (0.62, 0.66, 0.58, and 0.35) in the first year and at 40, 50, and 90 d (0.34, 0.21, and 0.30) in the second year. Finally, Figure 5d reveals a significant decrease in MDHAR activity at 40 and 50 d (0.29 and 0.25) in the first year and 50 d (0.11) in the second year for UV-B-treated samples. These results highlight the substantial impact of UV-B treatment on AsA-related enzymatic activities in mango fruit, reflecting changes in antioxidant mechanisms across the two growing seasons.

### 3.6. Transcriptome Analysis in Mango Under UV-B Stress

**Figure 6:**
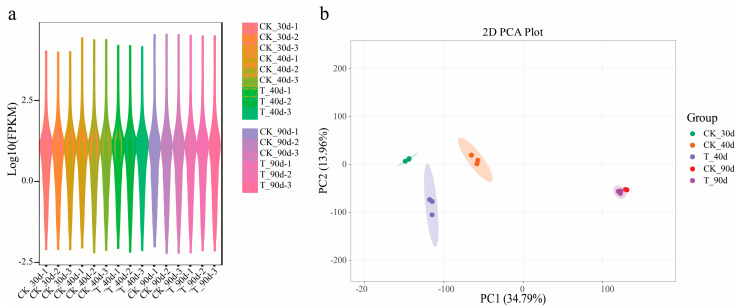
Sequence statistics of the transcriptome data: (**a**) Violin expression plot, different colors in the Figure represent different samples, and the width of each violin graphic reflects the number of genes at that expression level. (**b**) 2D principal component analysis (2D PCA), with different colors representing the control and treatment sample groups.

Transcriptome sequencing of mango provided crucial insights, with key RNA-seq properties summarized in Table S3. After thoroughly screening the raw data, a robust dataset with clean reads reaching 5 Gb was obtained for each sample. The overall base error rate was low at 0.01%, and the percentage of bases with Q20 and Q30 scores exceeded 98% and 94%, respectively, with a consistent GC content of 44–45%. These metrics confirm that the sequencing data were highly accurate and high-quality for further analysis. On average, over 70% of clean reads were uniquely aligned to the mango reference genome (Table S4). The distribution of gene expression across samples is illustrated with a violin plot in Figure 6a. Principal component analysis (PCA) of normalized counts was performed to assess the similarity of the datasets. PCA results indicated that samples within the same group, such as CK_40d, T_40d, and PC1, were clustered together, with 34.79% of the variation occurring between the groups (Figure 6b).

### 3.7. Analysis of DEGs in Mango Under UV-B Stress

**Figure 7:**
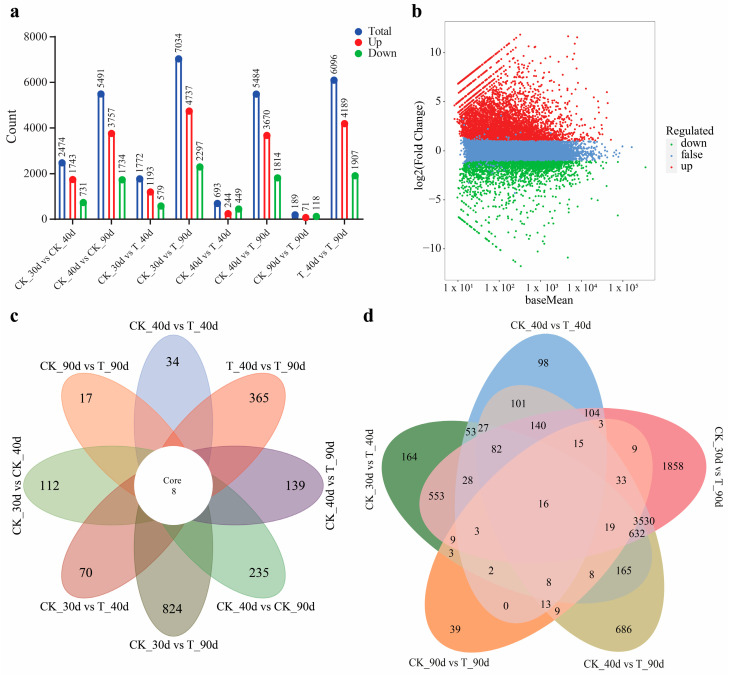
Overview of RNA-seq data: (**a**) statistics of gene expression across different conditions, (**b**) an MA plot showing differential gene expression of CK_30d vs. T_90d (**c**) a flower-shaped Venn diagram illustrating the relationships among differentially expressed genes (DEGs) across all eight groups, and (**d**) a Venn diagram depicting DEG relationships among the five specific groups.

Enhanced UV-B can induce significant changes in gene expression patterns. Therefore, DEGs among our sequenced samples were identified according to their differential expression levels. In eight comparisons (CK_30d vs. CK_40d, CK_30d vs. T_40d, CK_30d vs. T_90d, CK_40d vs. T_40d, CK_40d vs. CK_90d, CK_40d vs. T_90d, CK_90d vs. T_90d, and T_40d vs. T_90d), 2474, 1772, 7034, 693, 5491, 5484, 189, and 6096 DEGs were identified, respectively. In CK_30d vs. CK_40d, 1743 genes were upregulated and 731 downregulated. For CK_30d vs. T_40d, 1193 genes were upregulated and 579 downregulated. In CK_30d vs. T_90d, 4737 genes were upregulated and 2297 downregulated. CK_40d vs. T_40d had 244 upregulated and 449 downregulated genes. In CK_40d vs. CK_90d, 3757 genes were upregulated and 1734 downregulated, while CK_40d vs. T_90d showed 2708 upregulated and 2776 downregulated genes. CK_90d vs. T_90d had 71 upregulated and 118 downregulated genes. Finally, in T_40d vs. T_90d, 4189 genes were upregulated and 1907 downregulated. The CK_30d vs. T_90d comparison had the highest number of DEGs, highlighting significant transcriptomic changes due to UV-B stress at this stage. Conversely, CK_90d vs. T_90d showed a more stable transcriptome under UV-B conditions. Overall, more DEGs were upregulated than downregulated, except for CK_40d vs. T_40d and CK_90d vs. T_90d, suggesting that UV-B stress predominantly upregulates mango gene expression (Figure 7a).

An MA plot of CK_30d vs. T_90d illustrates these differential gene expression changes in Figure 7b, while the remaining comparisons are provided in the Supplementary Materials (Figure S1). To further explore the DEGs, we performed a flower plot analysis, visualizing gene expression patterns across all treatments as illustrated in Figure 7c. Each petal represents a comparison, showing the following DEG counts: CK_30d vs. CK_40d (112 DEGs), CK_30d vs. T_40d (70 DEGs), CK_40d vs. T_40d (34 DEGs), CK_40d vs. T_90d (139 DEGs), T_40d vs. T_90d (365 DEGs), CK_30d vs. CK_90d (235 DEGs), CK_90d vs. T_90d (23 DEGs), and CK_30d vs. T_90d (824 DEGs). The core of the flower plot highlights eight genes consistently differentially expressed across all conditions, indicating their key role in the plant’s response to UV-B stress.

A Venn diagram further dissects gene expression across five experimental conditions: CK_30d vs. T_40d, CK_40d vs. T_40d, CK_30d vs. T_90d, CK_90d vs. T_90d, and CK_40d vs. T_90d. Unique gene counts are as follows: CK_30d vs. T_40d (164), CK_40d vs. T_40d (98), CK_30d vs. T_90d (1858), CK_90d vs. T_90d (39), and CK_40d vs. T_90d (686) as illustrated in Figure 7c. A total of 16 genes were shared across all comparisons, while significant overlaps included 27 genes between CK_30d vs. T_40d and CK_40d vs. T_40d, 140 genes between CK_40d vs. T_40d and CK_40d vs. T_90d, and 553 genes between CK_30d vs. T_40d and CK_30d vs. T_90d. In total, 5530 genes were analyzed, with 2845 unique to specific conditions, highlighting the intricate gene expression patterns involved in mango’s response to UV-B stress, as depicted in Figure 7d. This analysis underscores the complexity and significance of both unique and shared gene expression patterns in elucidating underlying biological processes. This diagram effectively highlights both shared and unique elements across the five comparative conditions, providing a detailed visual representation of their relationships.

### 3.8. GO Enrichment Analysis of the DEGs in Mango Pulp Under UV-B Stress

**Figure 8:**
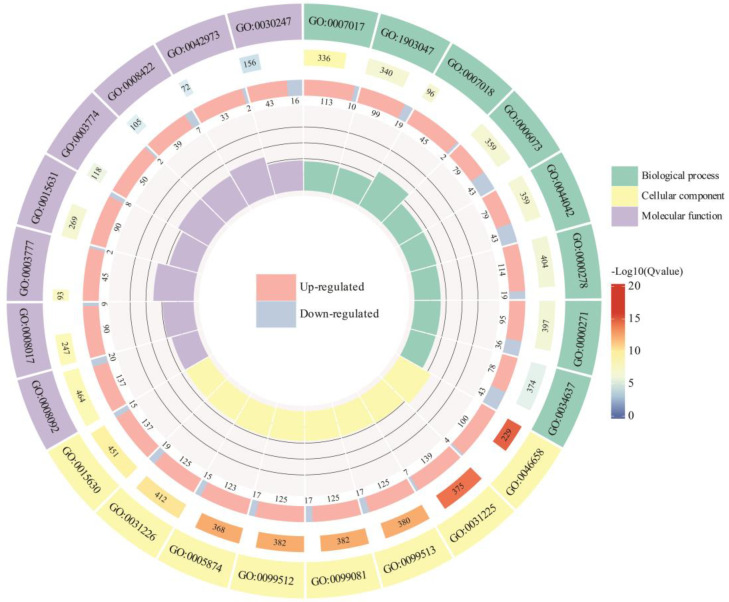
GO enrichment results of the DEGs in mango under UV-B stress CK_30d vs. T_90d based on q-value < 0.05. Top 20 GO enrichment results in mango.

To explore the functional roles of differentially expressed genes (DEGs) related to UV-B stress, we performed a Gene Ontology (GO) enrichment analysis using Q-values. Figure 8 illustrates the comparison between “CK_30d vs. T_90d”, highlighting the top 24 GO terms associated with DEGs. The outer ring of the circos plot lists these GO terms, each identified by a unique GO ID and categorized into three main types: biological processes, cellular components, and molecular functions. Significant biological processes in the “CK-30d vs. T_90d” comparison include microtubule-based processes (GO:0007017), mitotic cell cycle processes (GO:1903047), and cellular carbohydrate biosynthetic processes (GO:0034637). Key cellular components identified are anchored components of the plasma membrane (GO:0046658) and microtubules (GO:0005874). Important molecular functions include cytoskeletal protein binding (GO:0008092), microtubule binding (GO:0008017), and glucan endo-1,3-beta-D-glucosidase activity (GO:0042973). The middle ring of the plot shows the proportion of upregulated (blue) and downregulated (red) terms associated with each GO term, with segment lengths representing the number of genes. The innermost ring uses a color gradient from blue to red to indicate the significance of enrichment for each GO term. This visualization clearly compares the enriched functional categories between the two conditions, offering insights into the relevant biological processes, cellular components, and molecular functions. Additional comparisons can be found in Supplementary Figure S2.

### 3.9. KEGG Enrichment Analysis of the DEGs in Mango Pulp Under UV-B Stress

To better understand the pathway mechanisms associated with differentially expressed genes (DEGs), we conducted a KEGG enrichment analysis to identify significant metabolic pathways enriched with DEGs, using a *p*-value threshold of ≤0.05. We identified 20 KEGG pathways that were significantly enriched across the treatments. Several pathways showed significant enrichment in the UV-B vs. control comparison (CK_30d vs. T_90d). These include plant hormone signal transduction (ko04075), MAPK signaling pathway in plants (ko04016), starch and sucrose metabolism (ko00500), carbon metabolism (ko01200), glycolysis/gluconeogenesis (ko00010), pentose and glucuronate interconversions (ko00040), galactose metabolism (ko00052), biosynthesis of various plant secondary metabolites (ko00999), glutathione metabolism (ko00480), ascorbate and aldarate metabolism (ko00053), carotenoid biosynthesis (ko00906), terpenoid backbone biosynthesis (ko00900), flavonoid biosynthesis (ko00941), citrate cycle (TCA cycle) (ko00020), ribosome (ko03008), riboflavin metabolism (ko00740), flavone and flavonol biosynthesis (ko00944), fructose and mannose metabolism (ko00051), pentose phosphate pathway (ko00030), and isoflavonoid biosynthesis (ko00943). This analysis sheds light on the significant metabolic pathways in the UV-B stress response, offering insights into the underlying molecular mechanisms. Additionally, we focused on the glutathione and ascorbate pathways for further study. Detailed comparisons and results are available in Supplementary Figure S3.

**Figure 9:**
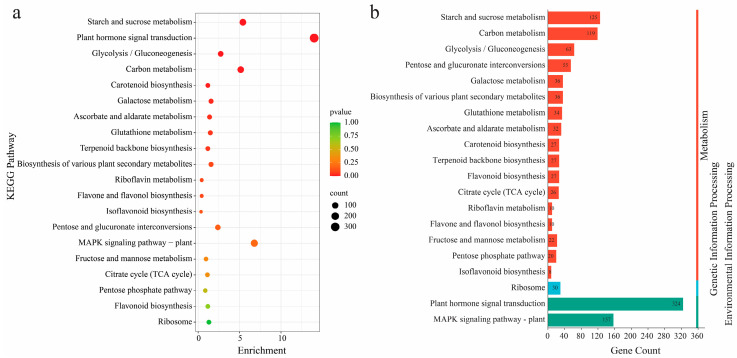
KEGG enrichment analysis of the DEGs across mango under UV-B stress: CK_30d vs. T_90d. (**a**) Enrichment represents the ratio between the number of DEGs mapped to a certain pathway that were significantly enriched based on *p*-value (FDR ≤ 0.05). (**b**) Total number of genes mapped to the corresponding pathway.

The secondary classification of pathways identifies 17 distinct subgroups within the metabolism pathway. These subgroups include starch and sucrose metabolism, carbon metabolism, glycolysis/gluconeogenesis, pentose and glucuronate interconversions, galactose metabolism, biosynthesis of various plant secondary metabolites, glutathione metabolism, ascorbate and aldarate metabolism, carotenoid biosynthesis, terpenoid backbone biosynthesis, flavonoid biosynthesis, citrate cycle (TCA cycle), riboflavin metabolism, flavone and flavonol biosynthesis, fructose and mannose metabolism, pentose phosphate pathway, and isoflavonoid biosynthesis (Figure 9a). A single subgroup, the ribosome, represents genetic information processing, while environmental information processing is divided into two subgroups: plant hormone signal transduction and MAPK signaling pathway in plants (Figure 9b). These classifications, observed across all treatments, offer a thorough view of the various biological processes and pathways involved in UV-B stress response. Additional details and comparisons are available in Supplementary Figure S4.

### 3.10. GSH Metabolism Pathway in Mango Under UV-B Stress

**Figure 10:**
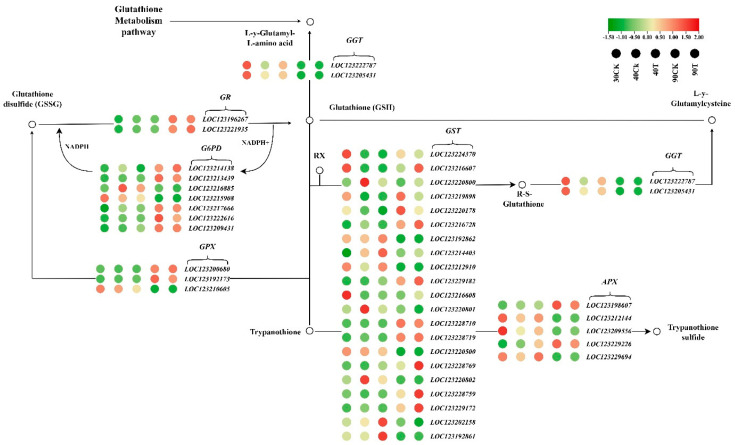
The KEGG metabolic pathway diagram illustrates the GSH metabolism pathway, highlighting the differentially expressed genes involved. Gene expression levels are indicated by color: upregulated genes are depicted in red, with darker shades indicating more significant upregulation, while downregulated genes are shown in green, with deeper shades representing more substantial downregulation.

To investigate the effect of UV-B treatment on mango pulp, 36 genes involved in the GSH metabolism pathway were analyzed from transcriptome data, and their expression levels were compared between UV-B-treated samples and controls over different periods. By the 40th day of UV-B treatment, the *MiGST* gene family displayed a shift, with six genes upregulated and nine downregulated, indicating a potential decrease in antioxidant capacity. The *MiGGT* family showed a slight upregulation in two genes, while the *MiGR* family continued to show moderate downregulation in two genes. In the *MiGPX* family, two genes remained downregulated, and the *MiG6PDH* family had four downregulated genes. On the 90th day of treatment, a more pronounced response was observed. The *MiGST* gene family had eight upregulated and seven downregulated genes, suggesting an enhanced antioxidant response. In the *MiGGT* family, two genes were slightly downregulated, while the *MiGR* family showed an upregulation in two genes. The *MiGPX* family had two upregulated genes and one downregulated gene, and in the *G6PDH* family, five genes were upregulated, and three were downregulated (Figure 10).

### 3.11. AsA Metabolism Pathway in Mango Under UV-B Stress

To investigate the impact of UV-B treatment on mango pulp, 23 genes related to the AsA metabolism pathway were screened from the transcriptome data, and their expression levels were compared with a control group. By the 40th day under UV-B treatment, two genes in the GalLDH gene family were upregulated. Similarly, two genes in the APX family and six in the AAO family also showed upregulated expression. The MDHAR family had one upregulated gene, while the DHAR gene family showed no expression. On the 90th day of treatment, one gene in the GalLDH gene family was upregulated, and two were downregulated. The APX family continued to show upregulation in two genes, but six genes in the AAO family were downregulated. Additionally, one gene in the MDHAR family and one gene in the DHAR family were downregulated.

**Figure 11:**
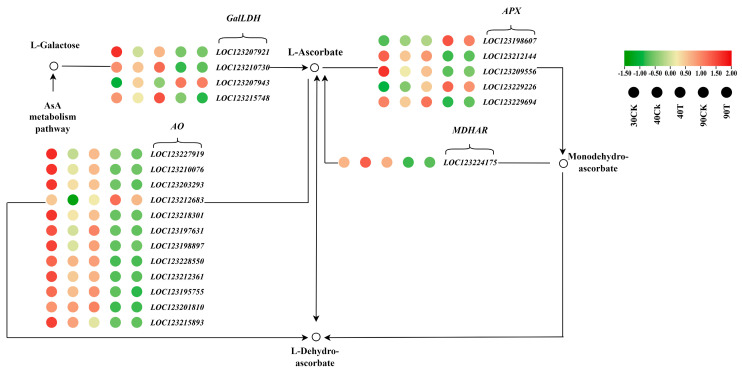
The KEGG metabolic pathway diagram illustrates the AsA metabolism pathway, emphasizing the differentially expressed genes involved. Gene expression levels are represented by color: upregulated genes are shown in red, with darker shades indicating more significant upregulation, while downregulated genes are depicted in green, with deeper shades reflecting more substantial downregulation.

UV-B treatment on mango pulp causes dynamic changes in the AsA metabolism pathway over time. Initially, GalLDH, APX, and AAO genes were strongly regulated by day 30, indicating an early adaptive response to UV-B stress. By 40 d, many genes remained upregulated but by 90 d, a more complex pattern appeared: GalLDH and APX genes were still upregulated, but AAO, MDHAR, and DHAR genes were downregulated. The downregulation of MDHAR and AAO genes at later stages may reflect a shift in the mango pulp’s metabolic priorities. As UV-B stress continues, the initial boost in antioxidant activity adjusts to a more balanced state, reducing the need for some antioxidant pathways. Despite this downregulation, the enhanced early activity of GalLDH, APX, and AAO genes helps maintain cellular redox balance and protect the pulp from UV-B-induced oxidative damage (Figure 11).

### 3.12. Real-Time PCR Validation

**Figure 12:**
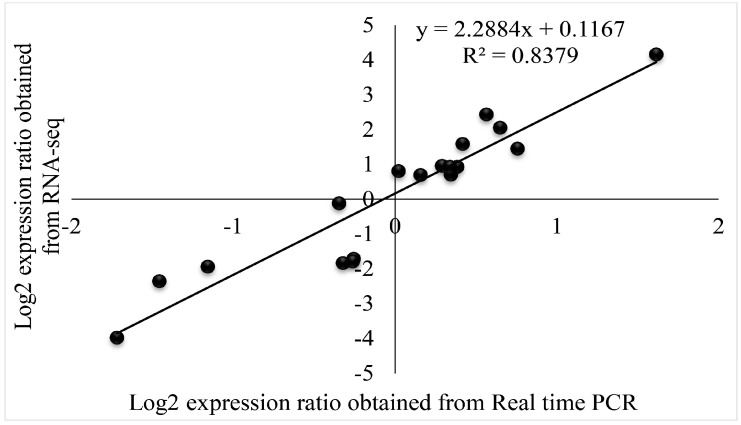
Coefficient analyses comparing gene expression levels from transcriptome sequencing (RNA-Seq) and real-time PCR data are shown. Scatterplots display the log2(expression ratios) for real-time PCR on the x-axis and RNA-Seq on the y-axis, based on the 2023 data.

To validate the RNA-seq data, eighteen genes were randomly selected for real-time PCR analysis, as shown in Figure 12. The results revealed a strong positive correlation (R^2^ = 0.8379) between the transcriptome data and real-time PCR results, confirming the reliability of the transcriptome data.

## 4. Discussion

### 4.1. Effect of ROS on Mango Under UV-B Stress

UV-B, an environmental light signal and abiotic stress factor, affects plant development dose-dependently [15]. At high doses, plants generate excessive oxygen stress, which damages DNA and proteins, triggering antioxidant systems to initiate defensive processes and mitigate damage [46]. The present study provides valuable insights into mango pulp’s oxidative stress and membrane integrity during ripening, particularly under UV-B radiation treatment. Our findings demonstrated that MDA levels increased under UV-B stress during both growing seasons, particularly at 90 d. This suggests enhanced lipid peroxidation during later developmental stages. While fluctuations were observed, the overall trend highlights increased oxidative damage with UV-B exposure in mango pulp, especially in the final stages of fruit development. This observation is consistent with previous reports associating elevated MDA levels with ROS-mediated oxidation of polyunsaturated membrane lipids and DNA [47,48]. The variations in MDA content observed during this study likely indicate early oxidative stress, with MDA accumulation resulting from ROS-induced lipid peroxidation in fruit tissues [49,50]. The increase in ion leakage observed in the first year suggests initial membrane damage followed by a stabilization period. In contrast, the ongoing rise in ion leakage during the second year points to sustained oxidative stress and continued membrane damage. These findings are consistent with previous research on lipid peroxidation and ROS-induced electrolyte leakage [51]. These results suggested that UV-B exposure consistently causes membrane damage throughout the fruit development stages. Compared to the control, H_2_O_2_ levels increased significantly in the first year under UV-B stress at 50 and 90 d, while a reduction was observed at 60 and 70 d, pointing to oxidative stress fluctuations throughout the season. H_2_O_2_ levels rose significantly at 60 and 90 d under UV-B stress but decreased at 50 d, indicating oxidative stress accumulation in later growth stages similar to the first year. In the second year, H_2_O_2_ levels increased significantly under UV-B stress at 50 and 90 d, while a reduction was observed at 60 and 70 d, indicating oxidative stress fluctuations throughout the season. H_2_O_2_ levels rose significantly at 60 and 90 d under UV-B stress but decreased at 50 d, indicating oxidative stress accumulation in later growth stages similar to the first year. This result aligns with previous literature suggesting that UV-B radiation induces ROS formation [52].

The •OH content rose significantly up to 60 d under UV-B treatment but decreased at 90 d, reflecting an early spike in oxidative stress followed by a decline during later developmental stages. In the second year, •OH levels spiked at 50 d but declined after 60 days, reflecting a transient increase in oxidative stress and a reduction toward the end of the growing season. Interestingly, hydroxyl ion content significantly decreased under UV-B treatment, which contrasts with studies in Vigna species that reported accelerated ROS generation, including •OH, under UV-B radiation, leading to lipid peroxidation and cellular damage [53]. This discrepancy suggests that different plant species may exhibit varying responses to UV-B radiation, potentially due to differences in their antioxidant defense mechanisms or the extent of oxidative stress they experience. Additionally, O_2_•^−^ levels increased significantly under UV-B stress at 80 d, indicating sustained oxidative stress and ROS production across most of the season. O_2_•^−^ content was substantially higher under UV-B stress at 90 d, indicating prolonged ROS accumulation throughout the later stages of fruit development, supporting the notion of oxidative stress under UV-B exposure [54]. However, it is worth noting that some studies have reported contrasting results, such as decreased singlet oxygen production in pre-irradiated tomatoes compared to controls [55]. Our findings show that UV-B radiation induces oxidative stress in mango pulp, generating ROS such as H_2_O_2_, •OH, and O_2_•^−^. This stress coincides with increased MDA levels, signaling membrane damage and lipid peroxidation. These results align with previous studies, illustrating the complex oxidative stress response in mangoes influenced by both UV-B exposure and fruit development stages. Prolonged UV-B exposure accelerated oxidative damage, compromising membrane stability and altering fruit development consistently over two years. Understanding these mechanisms is vital for optimizing postharvest management and improving mango storability.

### 4.2. Role of Non-Enzymatic Antioxidants on Mango Under UV-B Stress

The interaction between GSH and AsA is essential for the antioxidant defense mechanism in mango fruit. The AsA-GSH cycle is essential for the growth and development of fruit, underscoring the interconnected functions of these antioxidants in attenuating oxidative stress [56]. In our investigation, we monitored a steady rise in GSH levels during the early phases of treatment, especially when exposed to UV-B radiation exposure, followed by a subsequent decline at 90 d in both years. The observed pattern indicates that the accumulation of GSH is a fundamental component of the mango’s defensive mechanism during the initial phases of UV-B exposure [57,58]. This process is essential for maintaining cellular redox balance and mitigating oxidative stress. The rise in GSH levels during the early stages of fruit growth can be attributed to its role in neutralizing reactive oxygen species (ROS) and enhancing the antioxidant system through the AsA-GSH exchange cycle [59]. Nevertheless, there was a notable reduction in the concentration of GSH in mango fruit pulp subjected to UV-B radiation, as compared to the control.

This finding aligns with observations in pea plants [60], where GSH levels also declined under UV-B stress. Interestingly, our results contrast with reports of increased GSH content under UV-B treatment in other species, such as *Psoralea corylifolia* [61]. This discrepancy underscores the complexity of GSH responses to UV-B exposure, as different species may exhibit varied responses based on specific environmental conditions and the duration of exposure. For instance, ref. [62] observed a complex pattern of GSH response in plants exposed to low, chronic UV-B, highlighting the intricate nature of these antioxidant mechanisms. Regarding AsA content, our findings are consistent with previous studies suggesting that AsA content increased significantly after 60 d under UV-B stress. These findings suggest that mango fruit activates its antioxidative defenses during UV-B exposure, with fluctuations in GSH and AsA levels possibly reflecting shifts in oxidative stress management over time. This aligns with observations in broccoli plants [63] and other crops [64], where UV-B exposure has boosted AsA content. The increase in AsA under UV-B radiation highlights its crucial role in the antioxidant defense system, particularly in mitigating oxidative damage and maintaining fruit quality. Our study reveals a decrease in GSH content in mango fruit pulp under UV-B radiation, contrasting with reports of increased GSH levels in other plant species. This variation highlights the species-specific and dose-dependent nature of GSH responses to UV-B exposure. However, the observed increase in AsA content aligns with previous research, further emphasizing the complex and multifaceted nature of antioxidant responses in mango fruit. These findings underscore the importance of understanding species-specific antioxidant mechanisms to optimize fruit quality and resilience under environmental stress conditions. The contrasting trends in GSH and AsA levels—where GSH decreases and AsA increases highlight the intricate balance between these antioxidants in response to UV-B stress. Both are part of the AsA-GSH cycle, with GSH oxidizing to GSSG. The observed shift, where AsA accumulation is prioritized, may be a strategic response to the specific oxidative damage caused by UV-B. AsA plays a more prominent role in directly scavenging ROS or stabilizing other antioxidants [65]. This mechanism optimizes ROS detoxification and conserves critical antioxidants, which play a central role in mitigating UV-B-induced oxidative damage, providing valuable insights for future research on plant resilience to environmental stressors.

### 4.3. Dynamic Changes in GSH Metabolism Pathway in Mango Under UV-B Stress

The GSH-AsA metabolic cycle involves several enzymes and interconnected metabolic pathways. Under UV-B stress conditions, DEGs enriched by KEGG are primarily associated with *MiGGT*, *MiGR*, *MiGPX*, *MiGST*, *MiAPX*, and *MiMDHAR*. These genes play critical roles in regulating the activity of various components within the metabolic subsystems, which, together with transcriptional changes, ultimately affect the activity of GSH and AsA, contributing to the antioxidant capacity of plants. Glutathione reductase (GR), a key NADPH-dependent flavoprotein oxidoreductase, catalyzes the reduction of GSSG to GSH, protecting plant cells from oxidative damage caused by ROS bursts [66]. Studies have shown that *MiGR1* and *MiGR2* gene expressions and GR activity are significantly upregulated after 90 days of UV-B irradiation. Overexpression of chloroplast-localized *GR* in wheat and lettuce has also been observed to increase AsA and GSH levels during stress [67,68]. These findings indicate that the AsA-GSH cycle is vital for regenerating AsA and strengthening the protective response to UV-B radiation in mango pulp. Additionally, GGT enzymes are essential for handling oxidative stress by decomposing oxidized glutathione (GSSG). However, under extreme stress, such as heavy metal exposure or nutrient deficiencies, the increased demand for antioxidant defenses can lead to downregulating *MiGGT* gene expressions and enzyme activity, likely as a regulatory mechanism to balance the redox state and conserve resources [69,70]. Our study found that the expression levels of *MiGGT1* and *MiGGT2* genes and GGT enzyme activity were downregulated. The decreased activity of GGT, which is responsible for degrading GSH and breaking down glutathione conjugates, suggests a strategy to conserve GSH under stress. However, this conservation appears insufficient as GSH levels still decrease. Similarly, *OsGGT-3* and *OsGGT-1* were reported to be downregulated in response to heat stress [71]. Another study corroborates these findings, showing that both the expression of the *GGT1* gene and the associated enzymatic activity decreased under stress conditions [72]. GSTs play a critical role in detoxifying oxidative stress products by conjugating GSH with various toxic compounds [73], a function that becomes especially important under UV-B stress. This stress induces a significant increase in GST activity, as observed in various plants, including the upregulation of *MiGST1*, *MiGST2*, and *MiGST3* genes in our study. This heightened activity helps plants manage the elevated oxidative stress levels caused by UV-B exposure. For instance, in tomato plants, GST activity increases during salt stress, underscoring the enzyme’s role in stress response [74]. Additionally, studies have shown that UV-B radiation induces the expression of *GST* genes, such as *PcGST1* in parsley and *GSTU7* in other plants, within a few hours of exposure [75,76]. These findings highlight the integral role of GSTs in ROS scavenging and reducing oxidative damage, making them essential for plant survival under UV-B stress. GPXs are essential for scavenging ROS and protecting plant biomembranes from oxidative damage, crucial for growth and development [77]. ROS are generated by environmental stress and electron transfer chains in photosynthesis and respiration, underscoring the importance of GPXs in these processes. *GPX* expression levels affect plants’ H_2_O_2_ levels and oxidative stress [78,79]. Our study found an increase in GPX activity, particularly in the later stages of fruit development, highlighting its role in detoxifying elevated levels of H_2_O_2_ and lipid peroxides, thereby reducing oxidative damage during ripening [80]. This result aligns with other research, such as identifying GPX2 in Arabidopsis as a key gene in managing oxidative stress during late fruit ripening stages [81]. Likewise, our study’s upregulation of *MiGPX1* and *MiGPX2* suggests their essential role in scavenging ROS under abiotic stress, consistent with findings in Panax ginseng and sweet potato [82]. NADPH, a critical reductant molecule required in the AsA-GSH cycle involved in oxidative stress, is essential for maintaining cell GSH content. G6PDH is the major source of NADPH production. Under oxidative stress, the demand for NADPH increases to sustain a normal redox state, which may account for our study’s observed increase in G6PDH activity. Thus, we conclude that G6PDH plays a vital role in managing various oxidative stresses by providing a consistent supply of NADPH, thereby maintaining cell oxidative–reductive balance. In our study, we observed an increase in the expression levels of *G6PDH1* and *G6PDH2* genes, along with a corresponding rise in the enzymatic activity of G6PDH. This aligns with recent research that confirms *G6PDH’*s role in stress responses. For example, transgenic tobacco with the *PsG6PDH* gene improved cold tolerance [83]. Similarly, overexpressing *Cvcg6pdh* from Chlorella vulgaris boosted freezing tolerance in Saccharomyces cerevisiae, demonstrating G6PDH’s impact on freezing resistance [84]. G6PDH activity peaked at 90 h of stress exposure. This is consistent with findings from other studies, where G6PDH has been shown to play a key role in resistance to abiotic stress [85]. In Phragmites communis and wheat, G6PDH was essential in sustaining the GSH pool under salt stress [86].

### 4.4. Dynamic Changes in AsA Metabolism Pathway in Mango Under UV-B Stress

Multiple studies have demonstrated that the degradation of biosynthetic compounds and the subsequent recycling process, facilitated by enzymes and genes, may maintain the AsA levels within plant cells. AsA synthesis in plants is thought to be influenced by light through the galactose pathway, considered one of the most important pathways [87]. Our study showed that the galactose pathway had a major impact on AsA synthesis because of the rise in the enzymatic level and expression levels of GaIDH in mangos under UV-B irradiation. These findings support the contention that oxidative stress upregulated the expression level of GalLDH in Arabidopsis and tobacco [88,89]. As in the degradation process of AsA, two processes work together to maintain the level of AsA, which are APX and AO. Apx presented a peak-shaped trend in enzymatic acidity during growth under UV-B stress; however, there was also an upregulation trend in the gene expressions of APX. Our study found that APX is crucial in AsA accumulation in mango under UV-B and is critical in scavenging ROS. Similarly, exposure to barley drought conditions rapidly increases APX transcription and enzymatic activity, which enhances resistance to H_2_O_2_ in barley [90]. Guo et al. [91] demonstrated that *APX* gene expression is highly active in kiwi fruit leaves and roots, suggesting that increased enzymatic activity and gene expression are vital for regulating AsA content. Our study observed elevated expression of the *MiAPX1* and *MiAPX2* genes during the growth and development of mango fruit. AAO plays a crucial role in converting the redox state of AsA to its oxidized form, and regulating AAO gene expression is critical for both AsA metabolism and stress responses [92]. Our results revealed that AAO activity and gene expression levels were significantly downregulated after UV-B exposure in mango, suggesting that reduced AAO activity and expression contribute to AsA accumulation. A similar trend has been reported, where reducing AAO activity, which oxidizes AsA, may help maintain elevated AsA levels for effective ROS scavenging [93]. This aligns with prior studies on tomatoes, where suppression of AAO gene expression significantly increased AsA levels [94]. In this study, the significant downregulation of AAO4, AAO2, AAO3, and AAO4 gene expression and enzymatic activity at 90 days after UV-B irradiation corresponded with the observed AsA levels in mango. We hypothesize that this downregulation of AAO activity is part of a defensive response to sustain high AsA levels, mitigating increased H_2_O_2_ production in mangoes. Additionally, the AsA regeneration cycle plays a pivotal role in AsA accumulation. MDHAR, a key enzyme involved in AsA recycling, catalyzes the GSH-dependent reduction of dehydroascorbic acid to AsA, further supporting the role of this pathway in maintaining AsA levels under stress. MDHAR, which catalyzes MDHA to AsA, is crucial for AsA regeneration and maintaining the AsA pool. Our study found a decreased level and a downregulation trend in MDHAR, the gene encoding MiMDHAR. The downregulation of MiMDHAR points to a shift in prioritizing AsA accumulation over recycling its oxidized forms [95]. This is consistent with prior studies that demonstrated the suppression in the enzymatic activity of MDHAR gene expression in *Glycyrrhiza uralensis* and kiwi fruit [96,97].

Under UV-B stress, plants experience increased reactive oxygen species (ROS), necessitating a heightened antioxidant response. While the activity and gene expression of monodehydroascorbate reductase decreases, the ascorbate levels can paradoxically increase. This rise in AsA levels may be due to the plant’s compensatory mechanism to directly counteract ROS and maintain redox homeostasis when enzymatic defenses are compromised. The downregulation of MiMDHAR could result from oxidative damage to these enzymes or resource reallocation to other critical stress responses.

## 5. Conclusions

In conclusion, enhanced UV-B radiation significantly affects mango fruit quality and physiological responses over two growing seasons. Our findings suggest that UV-B treatment did not substantially affect fruit quality compared to the controls, but it did reduce fruit size. The study also highlights UV-B-induced oxidative stress, with elevated MDA, RC, H_2_O_2_, •OH, and O_2_•^−^ levels during mid-to-late growth, pointing to lipid peroxidation, ROS accumulation, and membrane damage. In response, mango fruit showed dynamic antioxidant changes: GSH levels rose early but declined later, while AsA levels increased during late stages. Key glutathione-related enzymes (*GR*, *GST*, *GPX*, and *G6PDH*) were elevated, while GGT activity decreased, reflecting a robust antioxidant defense. Ascorbic acid-related enzymes (*GalLDH* and *APX*) increased under UV-B, while AAO and MDHAR activities decreased. Gene expression analysis revealed upregulation of GSH metabolism genes (*MiGR*, *MiGPX*, *MiGST*, and *MiG6PD*) and AsA metabolism genes (*MiGalLDH* and *MiAPX*), while genes involved in GSH degradation (*MiGGT*) and AsA recycling (*MiAAO* and *MiMDHAR*) were downregulated. These findings provide insights into how UV-B radiation impacts mango development, quality, and antioxidant responses.

## Data Availability

The original contributions presented in the study are included in the article/Supplementary Material. Further inquiries can be directed to the corresponding author.

## Supplementary Materials

The following supporting information can be downloaded at https://www.mdpi.com/article/10.3390/antiox13111429/s1. Figure S1: MA plot showing differential gene expressions (a) CK_30d vs. CK_40d, (b) CK_30d vs. T_40d, (c) CK_40d vs. T_40d, (d) CK_40d vs. CK_90d, (e) CK_40d vs. T_90d, (f) CK_90d vs. T_90d, and (g) T_40d vs. T_90d. Figure S2: Gene Ontology (GO) enrichment results of the DEGs in mango under UV-B stress based on q-value < 0.05. Top 20 GO enrichment in mango. (a) CK_30d vs. CK_40d, (b) CK_30d vs. T_40d, (c) CK_40d vs. T_40d, (d) CK_40d vs. CK_90d, (e) CK_40d vs. T_90d, (f) CK_90d vs. T_90d, and (g) T_40d vs. T_90d. Figure S3: KEGG enrichment analysis represented the ratio between the number of DEGs mapped to a certain pathway that was significantly enriched in mango under UV-B stress based on p-value (FDR ≤ 0.05) (a) CK_30d vs. CK_40d, (b) CK_30d vs. T_40d, (c) CK_40d vs. T_40d, (d) CK_40d vs. CK_90d, (e) CK_40d vs. T_90d, (f) CK_90d vs. T_90d, and (g) T_40d vs. T_90d. Figure S4: KEGG enrichment analysis represented total number of genes mapped to the corresponding pathway in mango under UV-B stress (a) CK_30d vs. CK_40d, (b) CK_30d vs. T_40d, (c) CK_40d vs. T_40d, (d) CK_40d vs. CK_90d, (e) CK_40d vs. T_90d, (f) CK_90d vs. T_90d, and (g) T_40d vs. T_90d. Table S1: List of UV-B radiation intensities (kJ m^−2^ d^−1^) in natural light (10:00 a.m.–11:00 a.m.). Table S2: This table provides a list of 18 primers used for qRT-PCR. For each gene, the forward and reverse primer sequences are provided, along with the expected amplicon size in base pairs (bp) and melting temperature (Tm) of the primers. Table S3: Statistical results of transcriptome sequencing of mango fruit pulp data. Table S4: Statistical transcriptome sequencing results of mango fruit pulp data.
