# Supplementary material for: Glutathione and Ascorbic Acid Accumulation in Mango Pulp Under Enhanced UV-B Based on Transcriptome
Source: Antioxidants (Basel). 2024 Nov 20;13(11):1429. doi: 10.3390/antiox13111429 (PMC11591329; doi:10.3390/antiox13111429)
Supplement: Supplementary file 1 [file antioxidants-13-01429-s001.zip › Supplementary Figures.docx]

**Supplementary Figures**


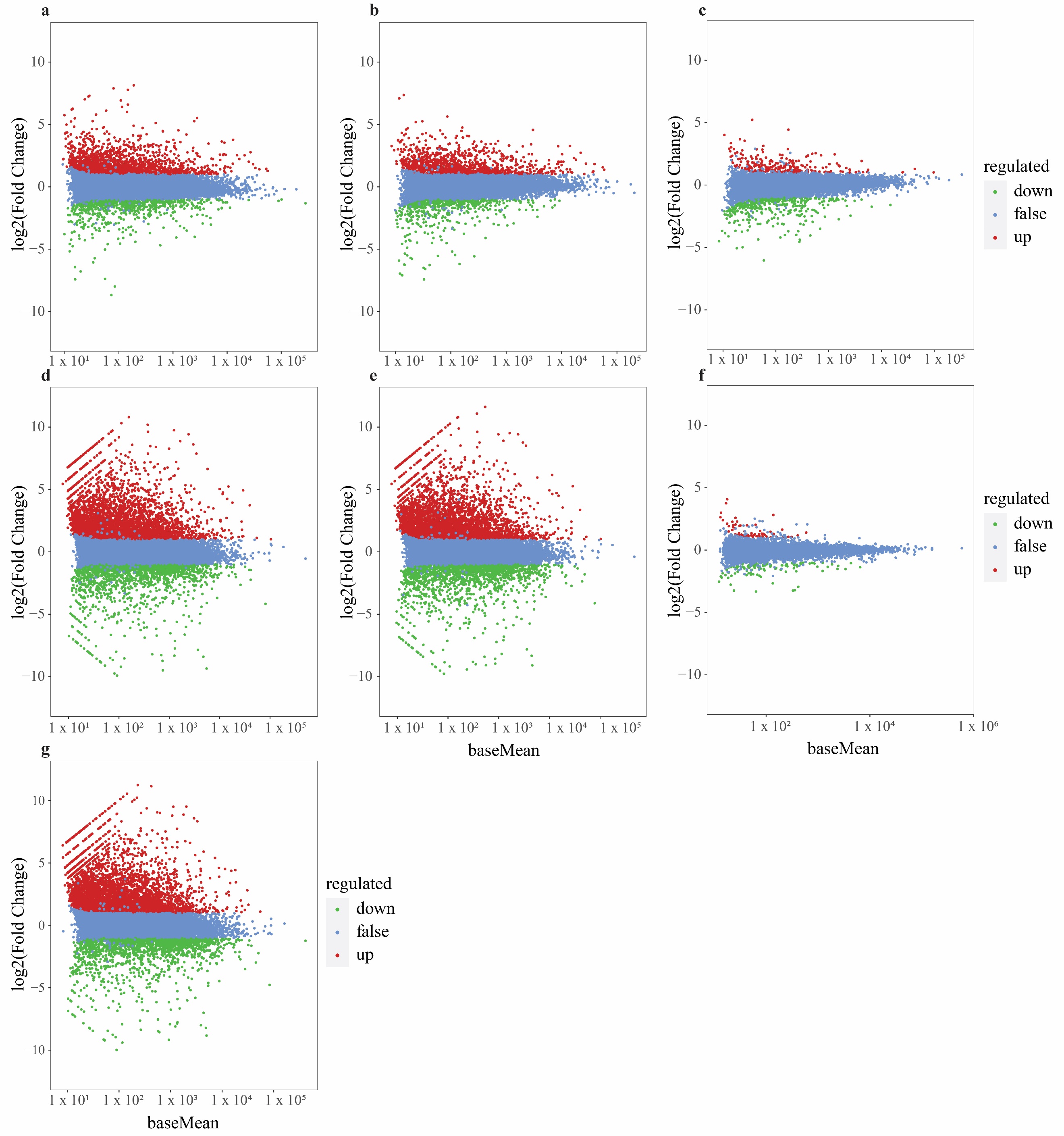


**Figure S1.** MA plot showing differential gene expressions (a) CK_30d vs CK_40d, (b) CK_30d vs T_40d, (c) CK_40d vs T_40d, (d) CK_40d vs CK_90d, (e) CK_40d vs T_90d, (f) CK_90d vs T_90d, and (g) T_40d vs T_90d.


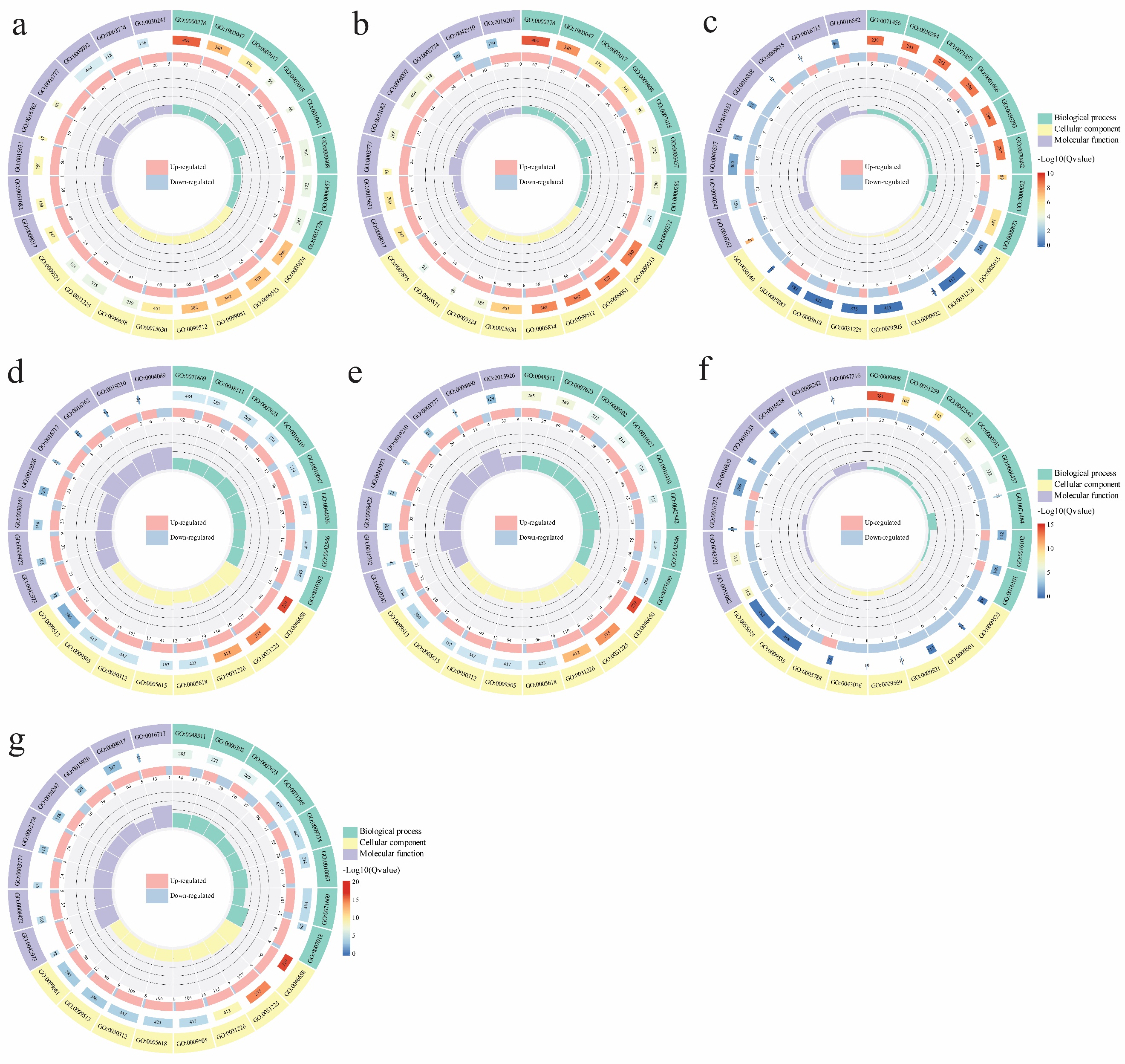


**Figure S2.** Gene Ontology (GO) enrichment results of the DEGs in mango under UV-B stress based on q-value <0.05. Top 20 GO enrichment in mango. (a) CK_30d vs CK_40d, (b) CK_30d vs T_40d, (c) CK_40d vs T_40d, (d) CK_40d vs CK_90d, (e) CK_40d vs T_90d, (f) CK_90d vs T_90d, and (g) T_40d vs T_90d.


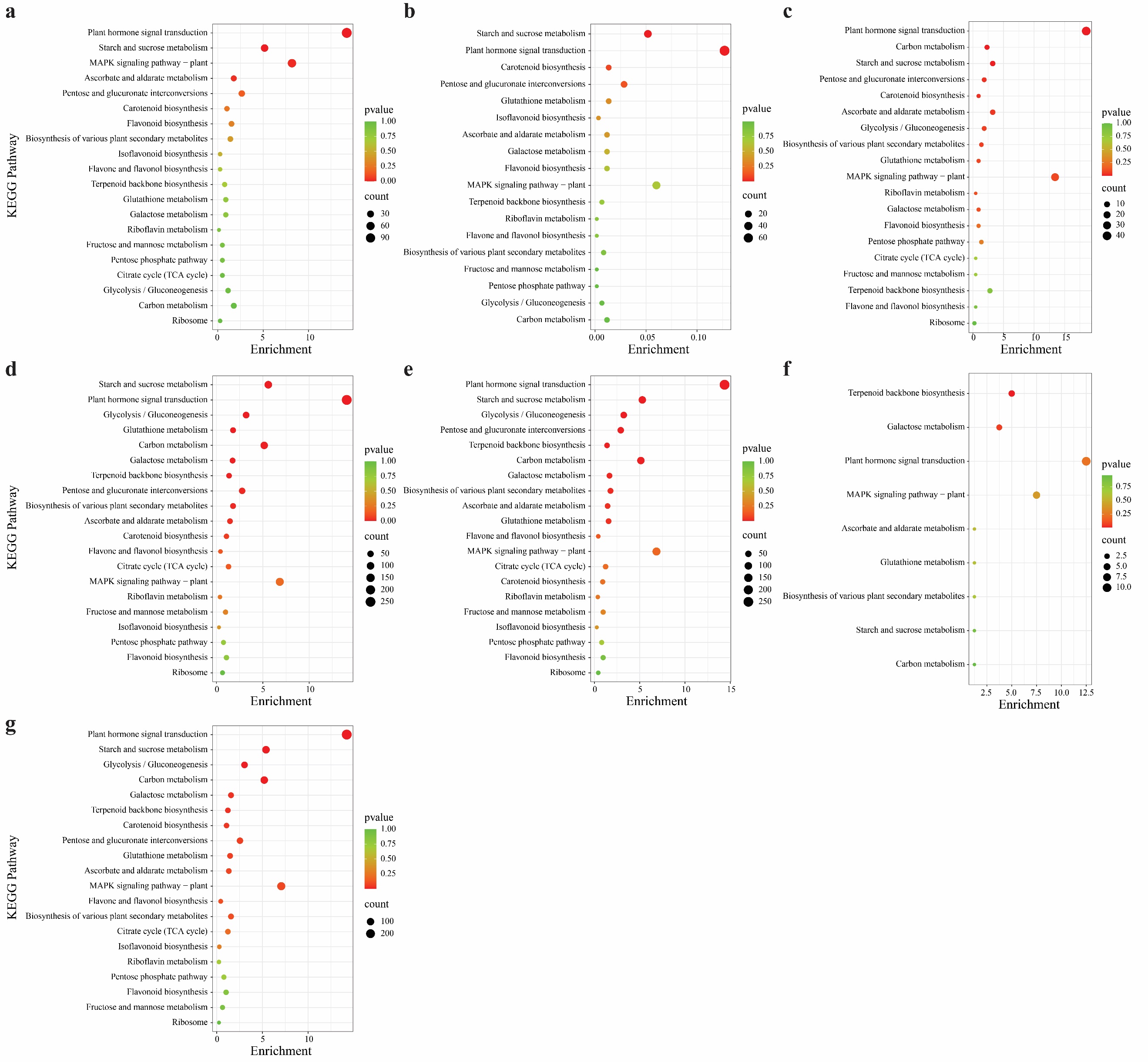


**Figure S3.** KEGG enrichment analysis represented the ratio between the number of DEGs mapped to a certain pathway that was significantly enriched in mango under UV-B stress based on p-value (FDR ≤ 0.05) (a) CK_30d vs CK_40d, (b) CK_30d vs T_40d, (c) CK_40d vs T_40d, (d) CK_40d vs CK_90d, (e) CK_40d vs T_90d, (f) CK_90d vs T_90d, and (g) T_40d vs T_90d.


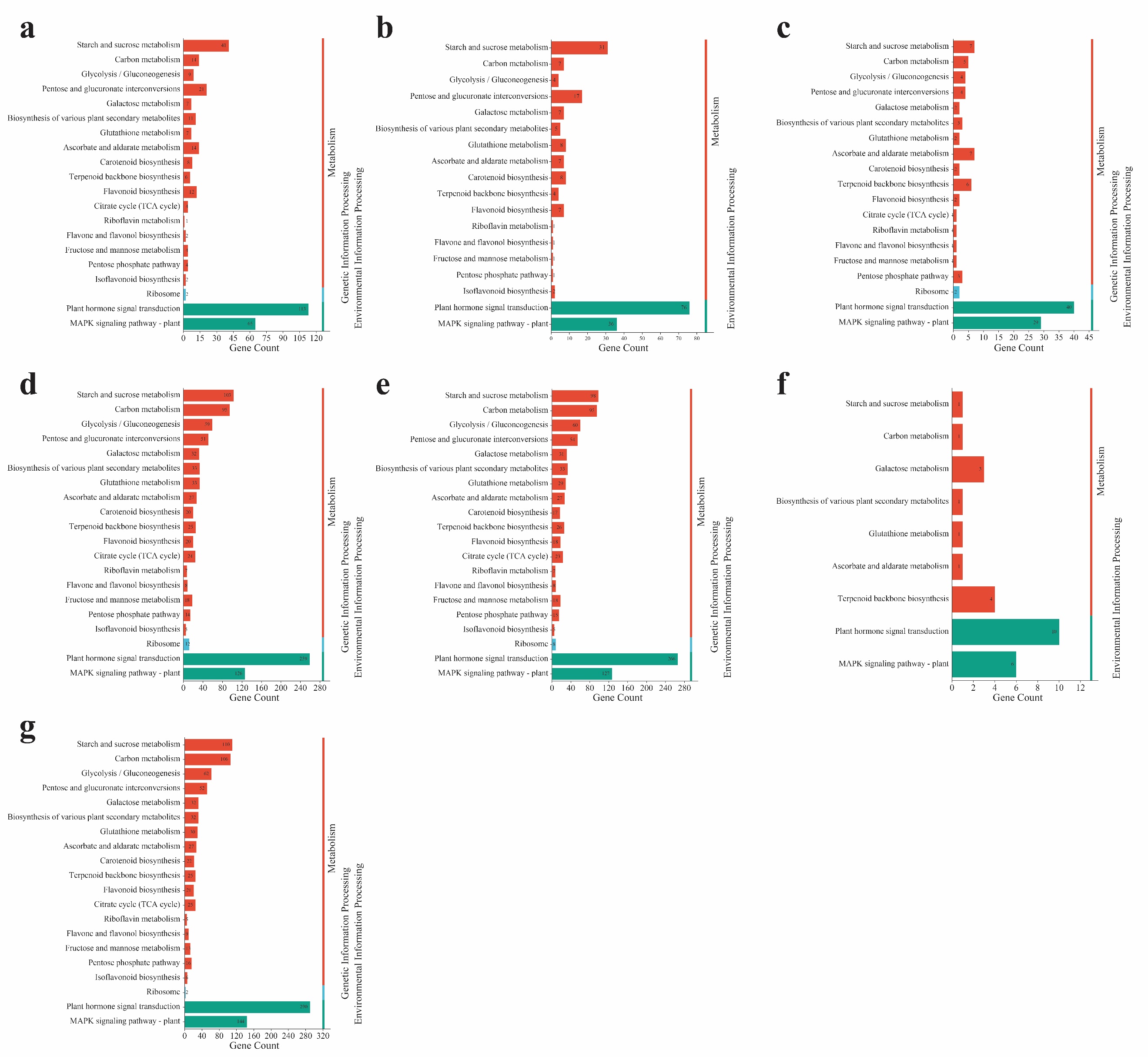


**Figure S4.** KEGG enrichment analysis represented total number of genes mapped to the corresponding pathway in mango under UV-B stress (a) CK_30d vs CK_40d, (b) CK_30d vs T_40d, (c) CK_40d vs T_40d, (d) CK_40d vs CK_90d, (e) CK_40d vs T_90d, (f) CK_90d vs T_90d, and (g) T_40d vs T_90d.
